# Effects of Hearing Intervention on Cognitive Function in Patients with Presbycusis: A Systematic Review and Meta-Analysis

**DOI:** 10.3390/audiolres16030067

**Published:** 2026-04-30

**Authors:** Yuxuan Li, Luofei Zhang, Jia Chen, Beibei Yang

**Affiliations:** 1 Second Affiliated Hospital, School of Medicine, Zhejiang University, Hangzhou 310009, China; 3180105284@zju.edu.cn (Y.L.); 22418485@zju.edu.cn (L.Z.); 2 Department of Otolaryngology, Second Affiliated Hospital, School of Medicine, Zhejiang University, Hangzhou 310009, China

**Keywords:** presbycusis, hearing intervention, cognition, hearing aids, cochlear implants

## Abstract

Introduction: This study aimed to systematically assess the impact of hearing interventions on cognitive function in older adults with presbycusis. Methods: A comprehensive search was conducted across PubMed, EMBASE, and Web of Science databases from their inception to 22 June 2025 to identify eligible randomized clinical trials or cohort studies that used designated cognitive scales or cognitive test measures. Two separate meta-analyses were conducted: one using uncontrolled pre–post comparisons and another restricted to studies that included concurrent untreated control groups. Results: A total of 22 studies were identified, comprising 9 focused on hearing aid use and 13 on cochlear implantation. Ultimately, 19 studies were included in the quantitative analysis: 7 on hearing aid use and 12 on cochlear implantation. The pooled analysis of hearing intervention across 17 studies involving 1562 patients indicated a 4% improvement in cognitive test scores post-intervention compared with pre-intervention (ratio of means: 1.04; 95% CI: 1.03–1.05; *p* < 0.001). However, in the 4 controlled studies that included an untreated comparator group (815 intervention, 7450 control participants), hearing intervention did not confer a statistically significant cognitive benefit over no intervention (SMD = 0.03; 95% CI: −0.04 to 0.09; *p* = 0.369). Conclusions: Current controlled evidence does not support the claim that hearing interventions preserve or enhance cognitive function in older adults with presbycusis.

## 1. Introduction

Presbycusis, also known as age-related hearing loss (ARHL), is defined as progressive bilateral sensorineural hearing loss (SNHL) that occurs as a result of the aging process in persons aged 50 years or older [1]. In 2019, approximately 1.57 billion people worldwide experienced hearing loss, accounting for one-fifth of the global population, with around 62.1% of these individuals being aged 50 or over [2]. As the global population ages rapidly, presbycusis has emerged as the third most prevalent chronic condition among older individuals [3], substantially reducing their quality of life and imposing significant social and economic burdens. Nevertheless, intervention rates for individuals with presbycusis remain low, with global hearing aid adoption rates standing at a mere 10–20% [4]. The reasons for this are multifaceted, with inadequate societal awareness of presbycusis being a key factor. On the one hand, individuals with presbycusis typically lack an understanding of the long-term implications of hearing impairment. Conversely, clinicians lack high-level, evidence-based medical guidance when determining treatment strategies. Beyond limited public awareness, stigma associated with hearing loss and hearing devices [5] and financial barriers [4]—including high out-of-pocket costs and lack of full insurance coverage—are major deterrents.

In recent years, the clinical importance of presbycusis has been amplified by evidence positioning it as an independent and modifiable risk factor for cognitive decline and dementia. Consequently, the 2020 Lancet Commission on Dementia Prevention, Intervention, and Care identified hearing loss intervention as the single most important modifiable risk factor for preventing dementia, suggesting that such interventions could effectively protect cognitive function [6]. This assertion has driven a surge of research into the cognitive benefits of hearing aids and cochlear implants.

Several mechanistic hypotheses have been proposed to explain the link between hearing loss and cognitive decline [7]. The cognitive load hypothesis suggests that degraded auditory signals force the brain to reallocate resources from higher-level cognitive processes (e.g., working memory and executive function) to auditory processing. This accelerates cognitive fatigue and decline. The sensory deprivation hypothesis suggests that chronic hearing loss leads to reduced social engagement and environmental stimulation, promoting brain atrophy and cognitive disuse. Alternatively, the common cause hypothesis argues that a shared neurobiological aging process (e.g., decline in central nervous system processing speed) underlies both hearing and cognitive deficits. Hearing interventions (e.g., hearing aids or cochlear implants) may exert their cognitive benefits through a combination of resource reallocation, enhanced social engagement, and preservation of neural integrity in auditory and cognitive networks.

Several studies suggest that hearing interventions, such as hearing aids or cochlear implants, may alleviate cognitive impairment in patients. For example, Mertens et al. [8] observed improvements in overall cognitive function and attention 14 months after cochlear implantation in patients with presbycusis. However, some studies have not demonstrated significant changes in cognitive function before or after intervention. Consequently, the precise impact of hearing interventions on cognitive function remains contentious. The existing evidence is predominantly derived from observational studies and non-randomized controlled trials with limited sample sizes, utilizing various cognitive assessment tools and producing highly heterogeneous outcomes.

Given this uncertainty, previous systematic reviews have largely relied on uncontrolled pre–post designs, which are susceptible to practice effects and other biases. The methodological innovation of this review is twofold: we conducted a pre–post meta-analysis and separately performed a meta-analysis restricted to studies with concurrent untreated control groups. By separating these two levels of analysis, we offer a more rigorous assessment of whether hearing interventions truly improve cognitive function in presbycusis.

## 2. Methods

This systematic review and meta-analysis followed the PRISMA statement (Supplementary Table S1) [9] and is registered with PROSPERO (CRD420251179437).

### 2.1. Literature Search Strategy

Eligible articles were identified through systematic literature searches. Databases, including PubMed, Web of Science, and Embase, were searched from inception to 22 June 2025.

Search terms: #1 (Presbycusis) OR (Age-related hearing impairment) OR (Age related hearing loss) OR (Older adults) OR (Elderly) AND #2 (cognitive) OR (cognition) OR (memory disorder) OR (alzheimer) OR (dementia) OR (mild cognitive impairment) OR (mini mental state examination) OR (mmse) OR (mini cog) OR (montreal cognitive assessment) OR (moca) OR (general practitioner assessment of cognition) OR (GPCOG) OR (clock drawing) OR (CDT) AND #3 (hearing intervention) OR (hearing interventions) OR (hearing aid) OR (hearing aids) OR (cochlear implant) OR (cochlear implants) OR (cochlear implantation) OR (hearing device) OR (hearing devices) OR (hearing treatment) OR (auditory implant) OR (auditory implants)

### 2.2. Inclusion and Exclusion Criteria

Inclusion criteria: (1) randomized clinical trials or cohort studies published as full articles involving participants aged 50 years or older with confirmed hearing loss through pure-tone audiometry; (2) hearing intervention utilizing hearing aids or cochlear implants; (3) assessment of global cognitive function using the Mini-Mental State Examination (MMSE) and/or Montreal Cognitive Assessment (MoCA), or evaluation of specific cognitive domains through common cognitive tests such as verbal and nonverbal measures (e.g., attention, delayed recall, fluency, immediate recall, processing speed, reasoning, semantic memory, visuospatial abilities, and working memory).

Exclusion criteria: (1) cross-sectional studies and those lacking hearing intervention or cognitive assessment post-intervention; (2) case reports, reviews, systematic analyses, conference papers, guidelines, posters, letters, and graduate theses; (3) duplicate studies from the same data source; (4) non-English language literature; (5) studies not utilizing any cognitive assessment tool (including scales and cognitive tests) for evaluating cognitive function.

### 2.3. Literature Quality Assessment and Risk of Bias Evaluation

Two researchers (Y.L. and L.Z.) independently evaluated the quality of studies and the risk of bias using the Newcastle Ottawa Scale (NOS) [10]. Any disagreements were resolved through discussion or, if consensus could not be reached, by the adjudication of a third reviewer (B.Y.). The assessment considered nine criteria, such as participant selection, comparability of intervention and control groups, and outcome measurement, with a maximum score of 9 points. Studies scoring 7–9 points were deemed to have a low risk of bias; those scoring 4–6 points were categorized as having a moderate risk of bias; and studies scoring 3 points or below were considered to have a high risk of bias. Moreover, when the number of included studies was 10 or more, publication bias was assessed using funnel plots and Egger’s test.

### 2.4. Data Extraction

Data extraction was conducted independently by two researchers. The extracted data included key characteristics of each eligible systematic review and meta-analysis, such as authors, publication year, country, study design, participant numbers, gender distribution, age range, follow-up duration, intervention modality, outcomes, cognitive evaluation methods, and primary conclusions. Furthermore, we extracted data pertaining to the assessment of effect size for cognitive outcomes following hearing interventions, which included the cognitive assessment tools utilized, as well as the mean and standard deviation of scores. In instances where studies did not report direct means and standard deviations (e.g., only median and 95% confidence intervals [CI] were provided), they were calculated according to the formula given in the Cochrane Handbook for Systematic Reviews of Interventions [11].

Due to the variety of cognitive assessment tools used in different studies, some of which evaluated different cognitive domains, the data extraction process categorized the tests used in each study by cognitive domain. These domains included global cognition, executive function, memory function, attention, information processing, and language. Different cognitive assessment tools and classifications are listed in Supplementary Table S2.

### 2.5. Statistical Analysis

We used the ratio of means (ROM) to evaluate changes in cognitive test scores pre- and post-application of hearing restoration devices. For studies that included a concurrent control group of participants with presbycusis who did not receive any hearing intervention, we performed a separate meta-analysis using the standardized mean difference (SMD) with 95% confidence intervals. This analysis was restricted to randomized controlled trials or cohort studies with an untreated comparator group to isolate the specific effect of hearing intervention from non-specific influences (e.g., practice effects).

All meta-analyses were performed using Stata 15 with a random-effects model due to anticipated heterogeneity in study designs, interventions, follow-up durations, and cognitive assessment tools. Cochrane’s Q and I^2^ statistics were used to test heterogeneity. If Q is statistically significant (*p* < 0.10), the I^2^ statistic estimates the percentage of sample differences due to heterogeneity. I^2^ values of 0–25% (very low), 25–50% (low), 50–75% (high), and 75–100% (very high) were used to classify heterogeneity levels. Furthermore, a sensitivity analysis was performed to evaluate the impact of excluding studies with atypical characteristics on heterogeneity and the overall pooled effect.

## 3. Results

**Figure 1:**
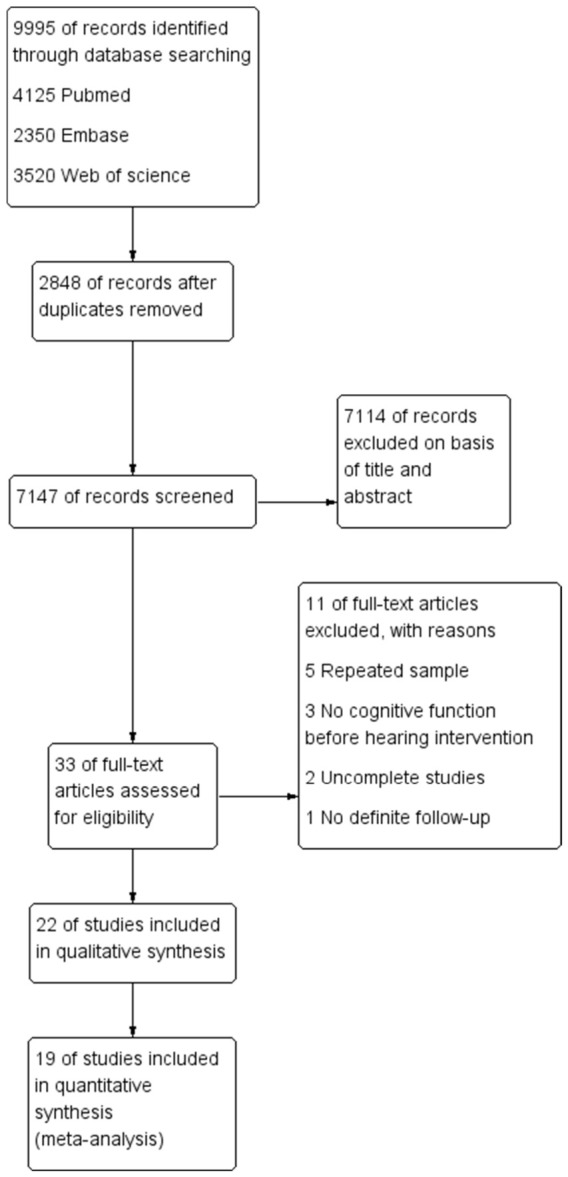
PRISMA flow diagram of the study screening process.

The PRISMA flow diagram illustrating the study screening process is presented in Figure 1. Following screening based on the specified criteria, 22 studies were included: 9 focused on hearing aids [12,13,14,15,16,17,18,19,20] and 13 on cochlear implants [21,22,23,24,25,26,27,28,29,30,31,32,33]. The fundamental characteristics of these 22 studies, including the first author, year, study design, sample size, age, gender, the type of hearing intervention, outcome measures, and NOS scale scores, are summarized in Supplementary Table S3. Among these studies, 14 were prospective cohort studies, 1 was a retrospective cohort study, 3 were randomized controlled clinical trials, 1 was a non-randomized clinical trial, and 3 were single-arm studies. According to the Newcastle–Ottawa Scale, 16 studies were classified as having a moderate risk of bias, while 6 were rated as having a low risk of bias (Supplementary Table S4).

In terms of cognitive domains, 18 of the 22 studies included evaluations of global cognition: 9 used the MMSE, and 3 utilized the MoCA. Additionally, 6 studies assessed executive function, 10 evaluated memory function, and 3 examined information processing, while 3 and 4 studies, respectively, investigated the attention and language domains.

Of the 19 quantitative studies, 17 provided sufficient pre–post data for within-subject comparisons, and 4 included a concurrent control group for a separate between-group meta-analysis.

### 3.1. Effect of Hearing Interventions on Cognitive Function

**Figure 2:**
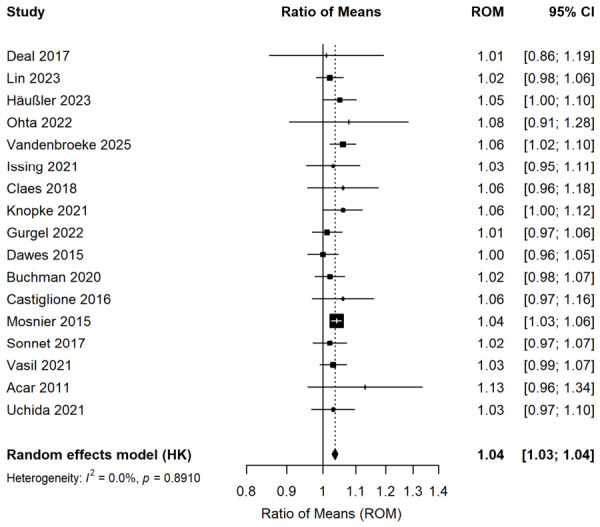
Forest plot of pre–post changes in cognitive test scores following hearing intervention (within-subject comparison, *n* = 1562). Squares represent effect sizes of individual studies (square size proportional to study weight); horizontal lines indicate 95% confidence intervals; diamonds represent pooled effect sizes.

A meta-analysis of cognitive test score changes pre- and post-hearing interventions from 17 studies (Figure 2) involving 1562 patients indicated a 4% improvement in cognitive test scores post-intervention compared to pre-intervention levels (ROM: 1.04; 95% CI: 1.03–1.05; *p* < 0.001). No significant heterogeneity was observed (I^2^ = 0%).

#### 3.1.1. Subgroup Analyses

**Figure 3:**
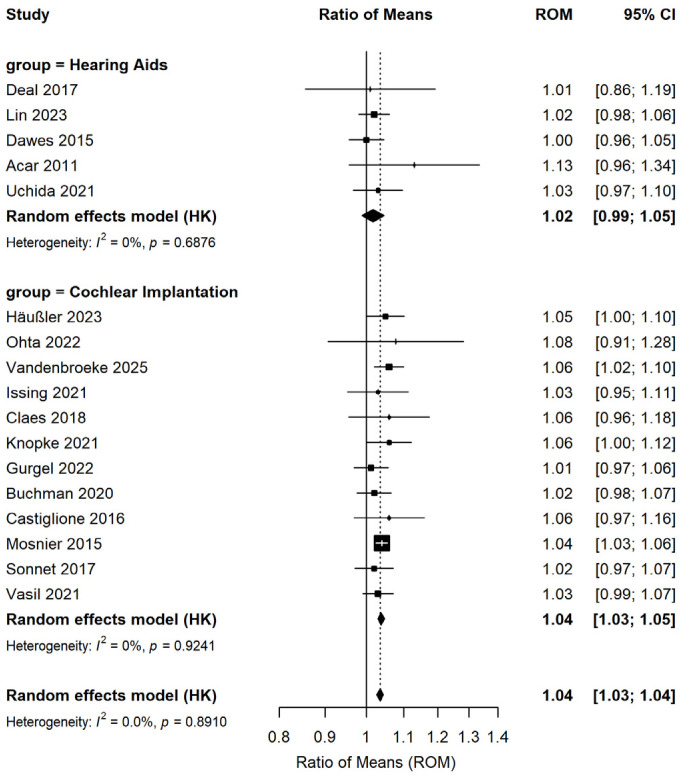
Forest plot showing the effect of hearing interventions on cognitive function stratified by intervention type. Squares represent effect sizes of individual studies (square size proportional to study weight); horizontal lines indicate 95% confidence intervals; diamonds represent pooled effect sizes.

**Figure 4:**
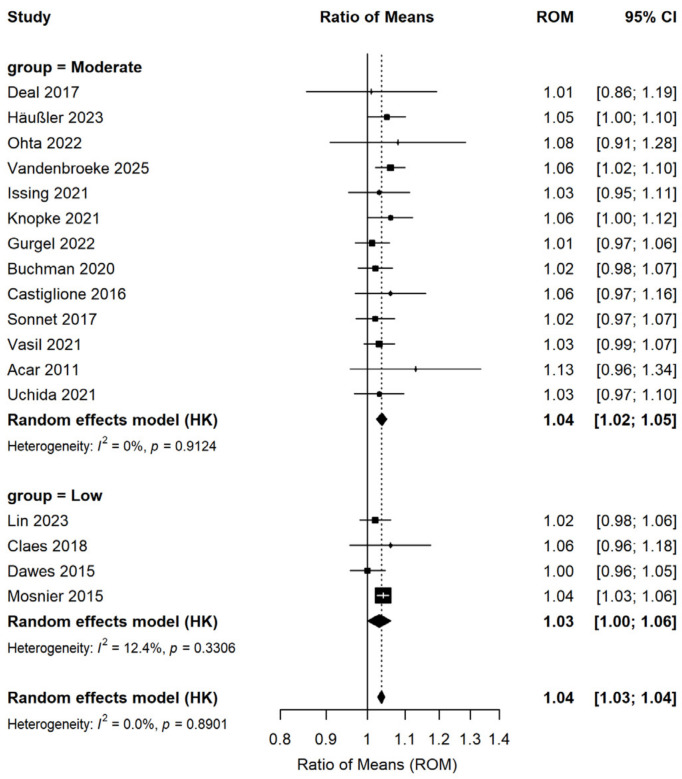
Forest plot of the effect of hearing interventions on cognitive function stratified by study quality. Squares represent effect sizes of individual studies (square size proportional to study weight); horizontal lines indicate 95% confidence intervals; diamonds represent pooled effect sizes.

**Figure 5:**
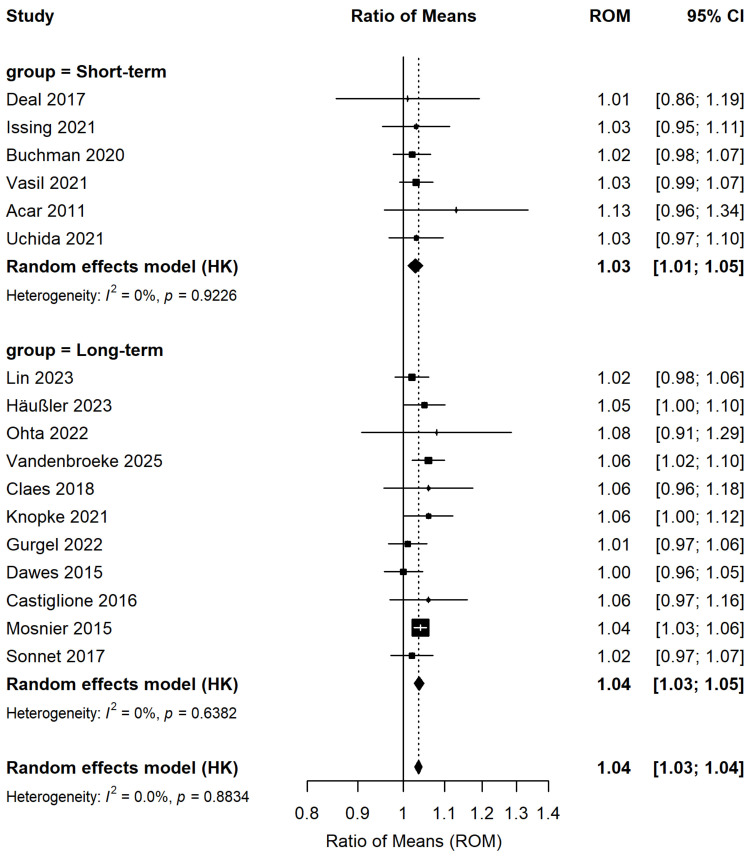
Forest plot of the effect of hearing interventions on cognitive function stratified by follow-up duration. Squares represent effect sizes of individual studies (square size proportional to study weight); horizontal lines indicate 95% confidence intervals; diamonds represent pooled effect sizes.

Subgroup analyses were performed according to the type of hearing intervention (Figure 3), study quality (Figure 4), and follow-up duration (Figure 5). Five studies involving 1076 patients assessed changes in cognitive test scores pre- and post-hearing aid usage, while 12 studies (486 patients) investigated cognitive function changes pre- and post-cochlear implantation. Results from subgroup analyses indicated significant enhancements in cognitive test scores for both the hearing aid subgroup (ROM: 1.02; 95% CI: 0.99–1.04; I^2^ = 0%) and the cochlear implant subgroup (ROM: 1.04; 95% CI: 1.03–1.05; I^2^ = 0%). Among the 17 studies, 4 exhibited a low risk of bias and 13 a moderate risk of bias. Subgroup analysis based on study quality demonstrated that both the low-risk-of-bias subgroup (ROM: 1.04; 95% CI: 1.02–1.05; I^2^ = 12.4%) and the moderate-risk-of-bias subgroup (ROM: 1.04; 95% CI: 1.03–1.05; I^2^ = 0%) displayed statistically significant improvements in cognitive test scores. To investigate whether the effect of hearing intervention on cognitive function differed by follow-up duration, we stratified the 17 studies into short-term (≤6 months, 6 studies) and long-term (>6 months, 11 studies) subgroups. The trend of improved cognitive test scores remained consistent in the short-term subgroup (ROM: 1.03; 95% CI: 1.00–1.05; I^2^ = 0%) and the long-term subgroup (ROM: 1.04; 95% CI: 1.03–1.05; I^2^ = 0%).

Publication bias was thoroughly evaluated through the utilization of funnel plots (Supplementary Figure S1) and quantitative assessment using Egger’s test. The results of the Egger test revealed no significant publication bias (intercept = 0.30, 95% confidence interval: −0.69–0.74, *p* = 0.93).

#### 3.1.2. Effects of Hearing Interventions on Different Cognitive Domains

We conducted a meta-analysis to assess changes in test scores across various cognitive domains before and after hearing interventions (Supplementary Figures S2–S6). The results indicated that hearing interventions resulted in varying degrees of improvement in test scores across cognitive domains, including global cognition (ROM: 1.04; 95% CI: 1.03–1.05; *p* < 0.001; I^2^ = 0%), executive function (ROM: 1.04; 95% CI: 0.98–1.10; *p* = 0.163; I^2^ = 0.7%), memory function (ROM: 1.05; 95% CI: 1.03–1.07; *p* < 0.001; I^2^ = 0%), information processing (ROM: 1.05; 95% CI: 1.02–1.08; *p* = 0.001; I^2^ = 0%), and attention (ROM: 1.07; 95% CI: 1.02–1.11; *p* = 0.003; I^2^ = 0%). Notably, improvements in cognitive test scores were statistically significant across all domains, except for executive function.

For the global cognition domain, publication bias was thoroughly evaluated using funnel plots (Supplementary Figure S7) and Egger’s test. The results of Egger’s test revealed significant publication bias (intercept = 0.02, 95% CI: −0.92 to 0.96, *p* = 0.04).

An analysis of the pooled effect, which involved sequentially excluding individual studies (Supplementary Figure S8), demonstrated low sensitivity to the inclusion of each study, thereby indicating the robustness of the findings.

### 3.2. Meta-Analysis of Controlled Studies

To address the potential confounding of practice effects and spontaneous cognitive decline, we performed a meta-analysis restricted to studies that included a concurrent control group. Four studies were included [15,16,19,20], comprising 815 participants in the intervention group and 7450 participants in the control group.

**Figure 6:**
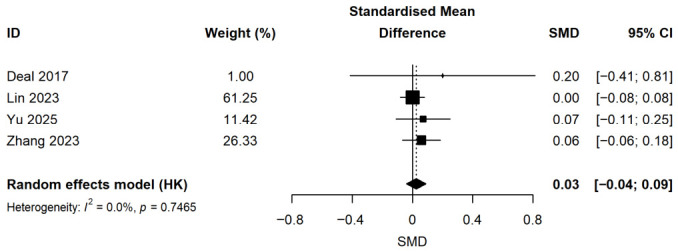
Forest plot of the effect of hearing intervention versus untreated control on cognitive function. Squares represent effect sizes of individual studies (square size proportional to study weight); horizontal lines indicate 95% confidence intervals; diamonds represent pooled effect sizes.

As shown in Figure 6, this result suggests that hearing intervention did not demonstrate a statistically significant advantage in improving cognitive test scores (SMD = 0.03; 95% CI: −0.04 to 0.09; *p* = 0.369) when compared with untreated control groups. No significant heterogeneity was observed (I^2^ = 0%).

Sensitivity analysis confirmed the robustness of this finding (Supplementary Figure S9).

### 3.3. Systematic Review

Three studies could not undergo quantitative analysis because pre- and post-intervention cognitive test scores could not be calculated or appropriately processed.

Amieva (2018) [13] conducted a prospective study involving 3777 participants aged 65 years and older, using a Cox proportional hazards model to assess the risk of dementia onset. The model indicated an increased risk of dementia among participants who self-reported hearing problems but did not wear hearing aids. Compared with those reporting no hearing problems, participants with hearing problems who used hearing aids showed no increased risk of dementia (HR = 0.86). Sarant (2024) [17] investigated 262 patients with age-related hearing loss (ARHL). After 36 months of hearing aid use, the hearing aid group (*n* = 160) maintained stable cognitive function, whereas the control group (*n* = 102) exhibited declines in working memory, visual attention, and psychomotor function. Völter (2023) [33] performed pre- and post-operative cognitive assessments on 75 elderly recipients of cochlear implants. The results revealed substantial improvements in cognitive function, specifically in recall and delayed recall, over five years.

## 4. Discussion

This systematic review and meta-analysis reveals two opposing sets of findings. On the one hand, an uncontrolled pre–post analysis of 17 studies comprising 1562 patients showed a 4% improvement in cognitive test scores after hearing intervention (ROM = 1.04; 95% CI: 1.03–1.05; *p* < 0.001). On the other hand, when the analysis was restricted to the four studies that included a concurrent untreated control group (815 intervention, 7450 control participants), hearing intervention did not confer a statistically significant cognitive advantage over no intervention (SMD = 0.03; 95% CI: −0.04 to 0.09; *p* = 0.369). This discrepancy is the central finding of our review and indicates that uncontrolled pre–post results may be heavily influenced by non-specific factors (e.g., practice effects, regression to the mean, patient expectancy, and increased test-taking confidence), whereas the controlled evidence does not support a specific cognitive-protective effect of hearing intervention.

The divergence between uncontrolled pre-post findings and controlled study findings may be explained by practice effects. Repeated administration of cognitive tests often leads to higher scores simply because of familiarity with test materials and procedures, independent of any true cognitive change [34]. This practice effect is well documented in older adult populations. In uncontrolled pre–post designs, practice effects cannot be separated from true intervention effects. Our controlled analysis, which compares intervention and untreated groups undergoing identical retesting schedules, can partially control for practice effects because any retest-related improvement would be expected in both groups. However, because practice effects might differ between groups (e.g., due to differential engagement or expectancy), the controlled analysis does not eliminate this source of bias. The null SMD (0.03; *p* = 0.369) suggests that the additional improvement specifically attributable to the hearing intervention is negligible. Therefore, the 4% pre–post improvement observed in uncontrolled analyses is largely, though not necessarily entirely, explainable by practice effects and other non-specific factors rather than by a genuine cognitive benefit of the device.

A second theoretical framework that deserves careful consideration is the common-cause hypothesis. The main mechanistic hypotheses linking hearing loss to cognitive decline—the cognitive load hypothesis [35] and the sensory deprivation hypothesis [7]—both predict that restoring auditory input should improve cognition. However, the controlled evidence from our meta-analysis does not support this prediction. An alternative explanation, the common-cause hypothesis [36], posits that both hearing loss and cognitive decline are manifestations of a shared underlying neurobiological aging process, such as a decline in central nervous system processing speed or generalized neural atrophy. Within this framework, a peripheral hearing device would not be expected to reverse or modify the common cause, and any observed cognitive changes would likely be non-specific or attributable to measurement artifacts. Our finding of no significant difference between the intervention and control groups is consistent with the common-cause hypothesis.

When interpreted cautiously, the pre–post improvements in specific cognitive domains and intervention subtypes still provide descriptive information. The pre–post data showed that attention improved most prominently (ROM = 1.07; 95% CI: 1.02–1.11; *p* = 0.003). This finding aligns with the theoretical framework proposed by Baltes and Lindenberger [37], who demonstrated that sensory functioning is more strongly coupled with fluid cognitive abilities (e.g., attention, processing speed, working memory) than with crystallized abilities. Attention, as a core component of fluid intelligence, is highly dependent on the integrity of sensory input. When auditory signals are degraded, attentional resources must be diverted to compensatory auditory processing, thereby depleting the capacity needed for sustained attention. By restoring auditory input, hearing interventions may reduce this compensatory demand, allowing attentional capacity to be reallocated to its primary function. Nevertheless, the four controlled trials included in our between-group meta-analysis did not report domain-specific data that would allow a separate analysis for attention. Therefore, whether the observed pre–post improvement in attention reflects a genuine intervention effect or is largely driven by practice effects, regression to the mean, or other non-specific factors remains unknown.

Executive function did not reach statistical significance in the pre–post analysis, and this negative finding is worth noting. The pooled analysis showed no significant pre–post improvement in executive function (ROM = 1.04; 95% CI: 0.98–1.10; *p* = 0.163). Several factors may explain this null result. First, executive function encompasses higher-order cognitive processes such as conflict management, inhibitory control, and decision-making [38], which may be less directly dependent on real-time sensory input and more influenced by educational background, cognitive reserve, and lifelong intellectual engagement. Second, some evidence suggests that older adults with age-related hearing loss and lower educational attainment show greater executive function improvements after cochlear implantation [39], indicating that baseline cognitive reserve may moderate the intervention effect. Third, the diversity of executive function assessment tools across studies may introduce differences in sensitivity that could attenuate the pooled effect size. Again, all these interpretations derive from pre–post analyses and await confirmation in controlled designs.

Publication bias in the cognitive domain analyses warrants attention. Egger’s test revealed significant publication bias across the cognitive domain analyses (intercept = 0.02; 95% CI: −0.92 to 0.96; *p* = 0.04), indicating an overrepresentation of studies reporting positive findings. Potential sources of publication bias include our restriction to English-language literature and the selective reporting of positive cognitive outcomes by original study authors. Consequently, the observed improvements in global cognition may be overestimated. Readers should interpret the domain-specific results with this bias in mind. Future systematic reviews should expand language and database coverage, and primary studies should prospectively register their protocols to mitigate selective outcome reporting.

Comparison with previous systematic reviews helps to contextualize our findings. Our results appear to diverge from some previous systematic reviews that reported positive cognitive effects of hearing interventions [40]. However, most prior meta-analyses relied predominantly on uncontrolled pre–post designs or observational studies with inherent biases, including practice effects and a lack of adequate comparators. Notably, the largest and most rigorously designed randomized controlled trial to date—the ACHIEVE trial [16]—found that hearing intervention did not reduce 3-year cognitive decline in its primary intention-to-treat analysis, although a benefit was observed in a subgroup of older adults from a different study population. Our controlled meta-analysis, which includes the ACHIEVE trial and three other controlled studies, corroborates this null finding. Therefore, despite the well-established benefits of hearing devices for communication, quality of life, and social functioning, the current evidence base does not support routine clinical recommendations that hearing intervention prevents or reverses cognitive decline in presbycusis.

This study has several limitations. First, most studies did not include patients with hearing loss without receiving hearing interventions as control groups, thereby constraining the ability to obviate practice effect issues and elucidate the treatment effects of hearing interventions on cognitive function in the presbycusis population. The four controlled studies that did include a comparator showed no significant intervention effect, but their number is small, and the control conditions varied. Furthermore, although this review categorized cognitive assessment tools by domain, variations in scoring criteria and sensitivity among the tools hinder direct comparisons of cognitive scores derived from different instruments across studies. Moreover, the follow-up durations across the included studies exhibited considerable variability, ranging from 3 months (Acar 2011 [12]) to 11 years (Dawes 2015 [14]). Half of the studies had follow-up periods shorter than 2 years, which limits the ability to comprehensively evaluate the long-term effects of hearing interventions on cognitive function. Short-term follow-up may also overlook the delayed effects of hearing interventions. Cochlear implant recipients need time to adjust to auditory input, and substantial cognitive improvements may manifest only after this adaptation period, which brief follow-ups may underestimate. Völter (2023) reported that patients demonstrated significantly enhanced cognitive function at the T3 follow-up compared with the T1 post-implantation assessment [33]. Similarly, research indicates that elderly cochlear implant recipients continue to display cognitive deficits relative to normal-hearing individuals one year after implantation [8]. Further clinical trials are necessary to determine whether hearing interventions can bridge this gap.

The implications for clinical practice and future research should be grounded in the limitations of the available evidence. Clinicians should recognize that while hearing devices improve auditory function and social engagement, the expectation that they preserve or enhance cognitive function is not currently supported by controlled evidence. Future research must prioritize large-scale, long-term randomized controlled trials with active control conditions (e.g., health education, social engagement, or auditory training without device provision), standardized cognitive outcome measures, and blinding of outcome assessors to isolate the true cognitive effects of hearing devices from non-specific influences.

While our review specifically focused on hearing devices (hearing aids and cochlear implants) as the primary means of audiological rehabilitation, it is important to acknowledge that psychosocial interventions (e.g., auditory training, communication strategies, or counseling) may also confer cognitive benefits [41]. These interventions might address cognitive decline through pathways distinct from sensory compensation, such as reducing social isolation or enhancing self-efficacy. Future studies should explore whether combining device-based interventions with psychosocial support yields synergistic effects on cognitive outcomes. Additionally, future studies should incorporate neuroimaging techniques, such as EEG [42] and functional MRI [43], to objectively record and quantify brain activity patterns and structural information. By analyzing activation patterns during auditory, language, and cognitive tasks, these methodologies could elucidate the neural basis of cognitive impairment in patients with presbycusis.

## 5. Conclusions

Uncontrolled pre–post analyses show a 4% improvement in cognitive test scores after hearing intervention in individuals with presbycusis. However, when compared with untreated control groups, hearing intervention does not confer a statistically significant cognitive advantage. This discrepancy suggests that the observed pre–post improvements may be attributable to non-specific influences—particularly practice effects—rather than a specific causal effect of hearing restoration. Therefore, the hypothesis that hearing intervention preserves or enhances cognitive function in older adults with presbycusis remains unsubstantiated by current controlled evidence. Large-scale, long-term randomized controlled trials with active control conditions and blinded outcome assessment are urgently needed to isolate the true cognitive effects of hearing devices from non-specific influences.

## Data Availability

Data in the manuscript were obtained from original publications.

## Supplementary Materials

The following supporting information can be downloaded at: https://www.mdpi.com/article/10.3390/audiolres16030067/s1, Table S1: PRISMA2020 checklist; Table S2: Testing tools used in the studies for different cognitive domains; Table S3:Characteristics of the included studies; Table S4: Risk of Bias Assessment (NOS); Figure S1: Funnel Chart of Changes in Cognitive Test Scores Before and After Hearing Intervention; Figure S2: Forest plot of changes in global cognitive test scores in global cognition before and after the hearing intervention (*n* = 535); Figure S3: Forest plot of changes in executive function test scores before and after hearing intervention (*n* = 151); Figure S4: Forest plot of changes in memory function test scores before and after hearing intervention (*n* = 1046); Figure S5: Forest plot of changes in attention domain test scores before and after hearing intervention (*n* = 80); Figure S6: Forest plot of changes in information processing domain test scores before and after hearing intervention (*n* = 87); Figure S7: Funnel plot of changes in global cognitive test scores before and after hearing intervention; Figure S8: Sensitivity analysis of global cognition test score changes before and after hearing intervention; Figure S9: Sensitivity analysis of cognition test score changes between intervention group and control group.

## Abbreviations

The following abbreviations are used in this manuscript:

ARHL	Age-related hearing loss
SNHL	Sensorineural hearing loss
MMSE	Mini-Mental State Examination
MoCA	Montreal Cognitive Assessment
NOS	Newcastle–Ottawa Scale
CI	Confidence interval
ROM	Ratio of means
SMD	Standardized mean difference
GC	Global cognition
EF	Executive function
MF	Memory function
IP	Information processing
