# Supplementary material for: Effects of Hearing Intervention on Cognitive Function in Patients with Presbycusis: A Systematic Review and Meta-Analysis
Source: Audiol Res. 2026 Apr 30;16(3):67. doi: 10.3390/audiolres16030067 (PMC13214493; doi:10.3390/audiolres16030067)

Supplementary Table S1 PRISMA2020 checklist

	Item #	Checklist item	Location where item is reported
**TITLE**			
Title	1	Identify the report as a systematic review.	1
**ABSTRACT**			
Abstract	2	See the PRISMA 2020 for Abstracts checklist.	1
**INTRODUCTION**			
Rationale	3	Describe the rationale for the review in the context of existing knowledge.	1
Objectives	4	Provide an explicit statement of the objective(s) or question(s) the review addresses.	2
**METHODS**			
Eligibility criteria	5	Specify the inclusion and exclusion criteria for the review and how studies were grouped for the syntheses.	3
Information sources	6	Specify all databases, registers, websites, organisations, reference lists and other sources searched or consulted to identify studies. Specify the date when each source was last searched or consulted.	3
Search strategy	7	Present the full search strategies for all databases, registers and websites, including any filters and limits used.	3
Selection process	8	Specify the methods used to decide whether a study met the inclusion criteria of the review, including how many reviewers screened each record and each report retrieved, whether they worked independently, and if applicable, details of automation tools used in the process.	3
Data collection process	9	Specify the methods used to collect data from reports, including how many reviewers collected data from each report, whether they worked independently, any processes for obtaining or confirming data from study investigators, and if applicable, details of automation tools used in the process.	3
Data items	10a	List and define all outcomes for which data were sought. Specify whether all results that were compatible with each outcome domain in each study were sought (e.g. for all measures, time points, analyses), and if not, the methods used to decide which results to collect.	6
	10b	List and define all other variables for which data were sought (e.g. participant and intervention characteristics, funding sources). Describe any assumptions made about any missing or unclear information.	6
Study risk of bias assessment	11	Specify the methods used to assess risk of bias in the included studies, including details of the tool(s) used, how many reviewers assessed each study and whether they worked independently, and if applicable, details of automation tools used in the process.	4
Effect measures	12	Specify for each outcome the effect measure(s) (e.g. risk ratio, mean difference) used in the synthesis or presentation of results.	4
Synthesis methods	13a	Describe the processes used to decide which studies were eligible for each synthesis (e.g. tabulating the study intervention characteristics and comparing against the planned groups for each synthesis (item #5)).	6
	13b	Describe any methods required to prepare the data for presentation or synthesis, such as handling of missing summary statistics, or data conversions.	4
	13c	Describe any methods used to tabulate or visually display results of individual studies and syntheses.	4
	13d	Describe any methods used to synthesize results and provide a rationale for the choice(s). If meta-analysis was performed, describe the model(s), method(s) to identify the presence and extent of statistical heterogeneity, and software package(s) used.	4
	13e	Describe any methods used to explore possible causes of heterogeneity among study results (e.g. subgroup analysis, meta-regression).	4
	13f	Describe any sensitivity analyses conducted to assess robustness of the synthesized results.	4
Reporting bias assessment	14	Describe any methods used to assess risk of bias due to missing results in a synthesis (arising from reporting biases).	4
Certainty assessment	15	Describe any methods used to assess certainty (or confidence) in the body of evidence for an outcome.	4

Section and Topic	Item #	Checklist item	Location where item is reported
**RESULTS**			
Study selection	16a	Describe the results of the search and selection process, from the number of records identified in the search to the number of studies included in the review, ideally using a flow diagram.	5
	16b	Cite studies that might appear to meet the inclusion criteria, but which were excluded, and explain why they were excluded.	5
Study characteristics	17	Cite each included study and present its characteristics.	6
Risk of bias in studies	18	Present assessments of risk of bias for each included study.	6
Results of individual studies	19	For all outcomes, present, for each study: (a) summary statistics for each group (where appropriate) and (b) an effect estimate and its precision (e.g. confidence/credible interval), ideally using structured tables or plots.	6
Results of syntheses	20a	For each synthesis, briefly summarise the characteristics and risk of bias among contributing studies.	6
	20b	Present results of all statistical syntheses conducted. If meta-analysis was done, present for each the summary estimate and its precision (e.g. confidence/credible interval) and measures of statistical heterogeneity. If comparing groups, describe the direction of the effect.	6
	20c	Present results of all investigations of possible causes of heterogeneity among study results.	6
	20d	Present results of all sensitivity analyses conducted to assess the robustness of the synthesized results.	9
Reporting biases	21	Present assessments of risk of bias due to missing results (arising from reporting biases) for each synthesis assessed.	9
Certainty of evidence	22	Present assessments of certainty (or confidence) in the body of evidence for each outcome assessed.	6
**DISCUSSION**			
Discussion	23a	Provide a general interpretation of the results in the context of other evidence.	10
	23b	Discuss any limitations of the evidence included in the review.	11
	23c	Discuss any limitations of the review processes used.	11
	23d	Discuss implications of the results for practice, policy, and future research.	12
**OTHER INFORMATION**			
Registration and protocol	24a	Provide registration information for the review, including register name and registration number, or state that the review was not registered.	
	24b	Indicate where the review protocol can be accessed, or state that a protocol was not prepared.	13
	24c	Describe and explain any amendments to information provided at registration or in the protocol.	13
Support	25	Describe sources of financial or non-financial support for the review, and the role of the funders or sponsors in the review.	13
Competing interests	26	Declare any competing interests of review authors.	13
Availability of data, code and other materials	27	Report which of the following are publicly available and where they can be found: template data collection forms; data extracted from included studies; data used for all analyses; analytic code; any other materials used in the review.	13

Supplementary Table S2 Testing tools used in the studies for different cognitive domains

**Cognitive domains**	**Measurement tools**
Global cognition （GC）	Mini-Mental State Examination (MMSE); The Montreal cognitive assessment (MoCA); Dementia detection test (DemTect); the CogState computerized Brief Battery Addenbrooke's Cognitive Examination III(ACE-III)；Wechsler Adult Intelligence Scale-Fourth Edition (WAIS-IV)
Executive function （EF）	Trial marking test (TMT-B, TMT-A); Digit Symbol Substitution Test (DSST); Stroop color word test (SCWT); Hayling Sentence Completion Test (Hayling)
Memory function （MF）	Digit span test (backward, forward); Delayed Word Recall Test (DWRT); Hopkins Verbal Learning Test Revised (HVLT-R); Spatial Span；Brief Visuospatial Memory Test-Revised (BVMT); Wechsler Adult Intelligence
Information processing （IP）	Wechsler Adult Intelligence Scale-IV-coding/symbol search (WAIS-IV-PSI); Digit coding; Digit Symbol task (DST)
Attention（AT）	D2 test
Language（LA）	Word fluency (F, A, S); Boston Naming Test

Supplementary Table S3 Characteristics of the included studies

study	country	study design	Patients （n）	follow up	age （mean）	Male n （%）	outcomes	Total Score /9
Intervention：Hearing Aids								
Acar 2011[12]	Turkey	Single Arm Trial	34	3 months	70.08	30（88.2）	GC(MMSE)	5
Amieva 2018*[13]	France	Prospective Cohort	3588	3 years	75.3	1514 （42.2）	GC(MMSE)	8
Dawes2015[14]	USA	Prospective Cohort	666	11years	68.2	491 （73.7）	GC (MMSE)	7
Deal 2017[15]	USA	Randomized Controlled Trial	40	6 months	77.5	8（65）	EF (TMT， DSST), MF (Delayed word recall, Logical memory A, Incidental learning), LA (Word fluency F, A, S；Boston Naming Test)	7
Lin 2023[16]	USA	Randomized Controlled Trial	977	3 years	76.8	454 （46.5）	EF (TMT A and B, DSST), MF (delayed word recall, logical memory, incidental learning), LA (BNT, word fluency, animal naming),	8
Sarant 2024*[17]	Germany	Prospective cohort	262	36 months	73.9	127(58.5)	GC (MMSE， The CogState Brief Battery)	7
Uchida 2021[18]	Japan	Prospective Cohort	94	6 months	76.9	39（41.5）	EF (DSST)	7

study	country	study design	Patients （n）	follow up	age （mean）	Male n （%）	outcomes	Total Score /9
Yu 2025[19]	UK	Randomized Controlled Trial	58	6 months	78.85	20(34.5)	GC (ACE-III), EF (TMT-A, TMT-B), MF (memory, DWRT)	7
Zhang 2023[20]	China	Prospective Cohort	30897	7 years	NR	13094(42.4)	GC (MMSE)	8
Intervention：Cochlear Implantation								
Buchman 2020[21]	USA	Non-Randomized Controlled Trial	96	6 months	71	62（65）	GC (MoCA)	7
Castiglione 2016[22]	Italy	Prospective Cohort	15	1 year	71	8（53.3）	GC (MoCA)	6
Claes 2018[23]	Belgium	Prospective Cohort	20	1 year	71.5	12（60）	GC (RBANS-H), MF (RBANS-H domains-the immediate memory, Visuospatial/constructional and delayed memory), AT (RBANS-H domain-attention), LA (RBANS-H domain-language)	8

study	country	study design	Patients（n）	follow up	age（mean）	Male n（%）	outcomes	Total Score /9
Gurgel 2022[24]	USA	Prospective Cohort	48	1 year	79.4	32(86)	GC (MMSE), EF (TMT A and B, SCWT, Hayling), MF (Digit Span, HVLT-R, Spatial Span, BVMT), AT (d2 Test)	5
Häußler 2023[25]	Germany	Single Arm Trial	33	2 years	75.5	19(57.6)	WAIS-IV	5
Issing 2021[26]	Germany	Prospective Cohort	41	6 months	64	——	GC (DemTect), EF (TMT), MF (DemTect-word list, DemTect-digit span, DemTect-delayed recall), IP (transcoding)	5
Knopke 2021[27]		Prospective Cohort	21	1 year	77.1	11(52.4)	GC (WAIS-IV), MF (WMI-Digit Span and Arithmetic), IP (PSI-Symbol Search and Coding)	6
Mosnier 2015[28]	France	Prospective Cohort	94	1 year	72	45（43.9）	GC (MMSE)	8
Ohta 2022[29]	Japan	Prospective Cohort	21	2 years	median:69	7(33.3)	GC (MMSE)	6
Sonnet 2017[30]	France	Prospective Cohort	16	1 year	72.5	6（37.5）	GC (MMSE)	7

study	country	study design	Patients（n）	follow up	age（mean）	Male n（%）	outcomes	Total Score /9
Vandenbroeke 2025[31]	Belgium	Prospective Cohort	25	4 years	69.8	16(64)	GC (RBANS-H), MF (RBANS-H domains-Immediate memory, Visuospatial/Constructional, Delayed memory), AT (RBANS-H domain-attention), LA (RBANS-H domain-language)	5
Vasil 2021[32]	USA	Single Arm Trial	77	6 months	72.4	53（68.8）	GC (MoCA)	6
Völter 2023*[33]	USA	Retrospective Cohort	75	5 years	65.41	30(40)	MF (Immediate and delayed recall)	5

***Studies not included in the quantitative analysis.**

The full names of cognitive assessment tools listed only by their English abbreviations are provided in Supplementary Table 1.

Supplementary Table S4 Risk of Bias Assessment（NOS）

First Author, Year	Representative of Exposed Cohort	Selection of Non Exposed Cohort/Controls	Exposure	Outcome of Interest Not Present at The Start of The Study?	Other Co Morbidities Compared?	Age & Education?	Assessment of Outcomes by Record Linkage?	Minimum 30-Day Follow-Up	Complete Follow-Up	Total Score /9	Risk of Bias (*8-9 scores: Low, 5-7 scores: Moderate, 0-4 stars: High)
Acar 2011[12]	0	0	1	1	0	0	1	1	1	5	Moderate
Amieva 2018*[13]	1	0	1	1	1	1	1	1	1	8	Low
Dawes2015[14]	1	0	1	1	1	1	1	1	1	8	Low
Deal 2017[15]	1	1	1	1	1	0	1	1	0	7	Moderate
Lin 2023[16]	1	1	1	1	1	1	1	1	0	8	Low
Sarant 2024*[17]	0	0	1	1	1	1	1	1	1	7	Moderate
Uchida 2021[18]	1	0	1	1	1	1	1	1	0	7	Moderate
Yu 2025[19]	1	1	1	1	1	0	1	1	0	7	Moderate
Zhang 2023[20]	1	1	1	1	1	1	1	1	0	8	Low
Buchman 2020[21]	1	1	1	1	1	0	1	1	0	7	Moderate
Castiglione 2016[22]	0	1	1	1	0	0	1	1	1	6	Moderate
Claes 2018[23]	1	0	1	1	1	1	1	1	1	8	Low
Gurgel 2022[24]	0	0	1	1	1	0	1	1	0	5	Moderate
Häußler 2023[25]	0	0	1	1	1	0	1	1	0	5	Moderate
Issing 2021[26]	0	0	1	1	0	0	1	1	1	5	Moderate

First Author, Year	Representative of Exposed Cohort	Selection of Non Exposed Cohort/Controls	Exposure	Outcome of Interest Not Present at The Start of The Study?	Other Co Morbidities Compared?	Age & Education?	Assessment of Outcomes by Record Linkage?	Minimum 30-Day Follow-Up	Complete Follow-Up	Total Score /9	Risk of Bias (*8-9 scores: Low, 5-7 scores: Moderate, 0-4 stars: High)
Knopke 2021[27]	0	0	1	1	1	0	1	1	1	6	Moderate
Mosnier 2015[28]	1	0	1	1	1	1	1	1	1	8	Low
Ohta 2022[29]	0	0	1	1	1	0	1	1	1	6	Moderate
Sonnet 2017[30]	1	0	1	1	1	1	1	1	0	7	Moderate
Vandenbroeke 2025[31]	0	0	1	1	1	1	0	1	0	5	Moderate
Vasil 2021[32]	1	0	1	1	1	0	1	1	0	6	Moderate
Völter 2023*[33]	0	0	1	1	1	1	0	1	0	5	Moderate

Supplementary Figure S1 Funnel Chart of Changes in Cognitive Test Scores Before and After Hearing Intervention

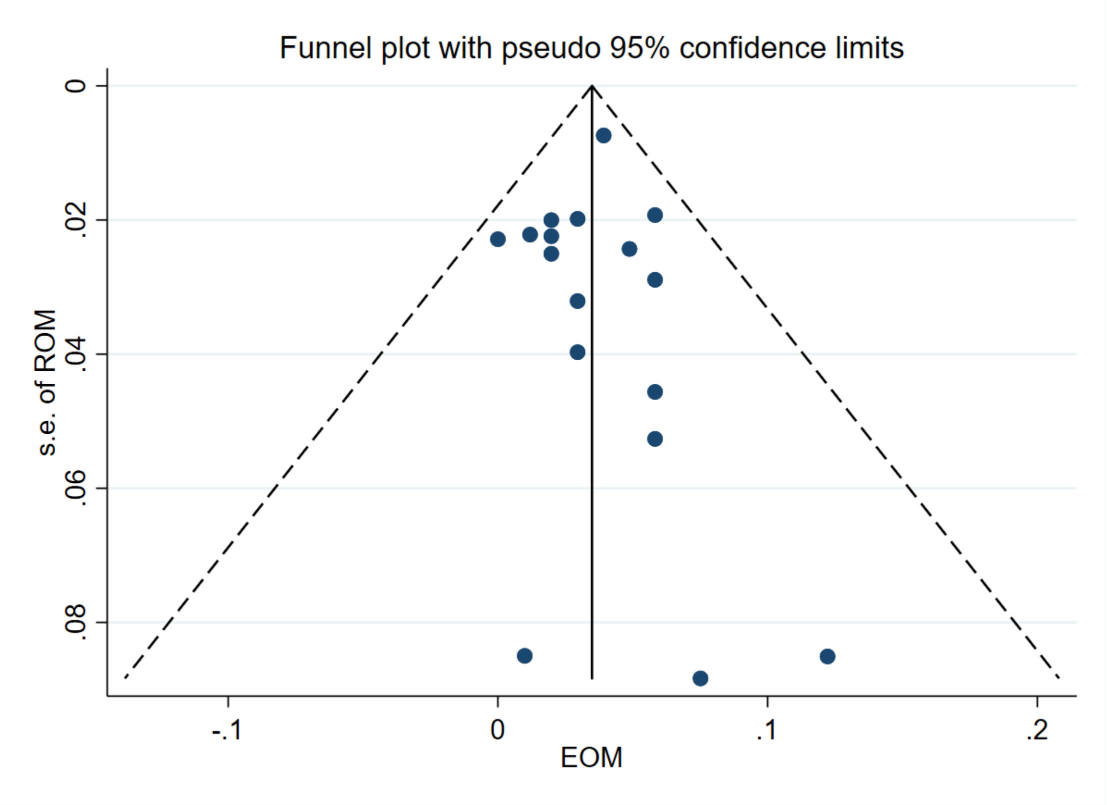

Supplementary Figure S2 Forest plot of changes in global cognitive test scores in global cognition before and after the hearing intervention (n=535)

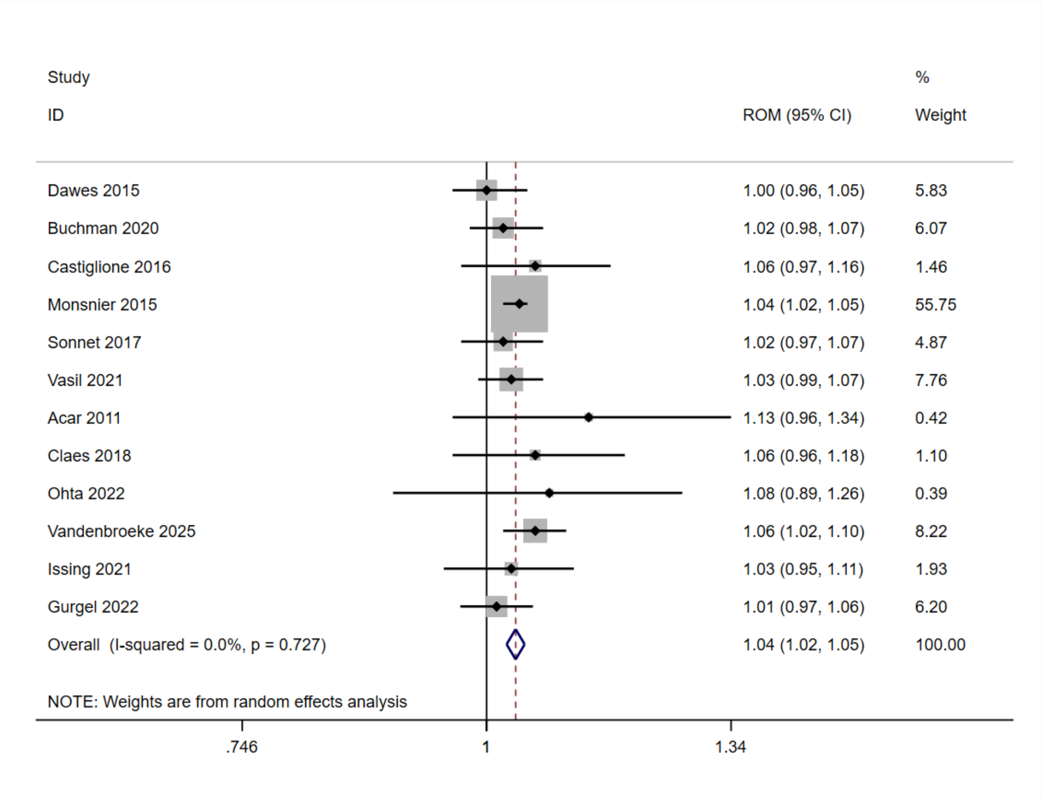

Supplementary Figure S3 Forest plot of changes in executive function test scores before and after hearing intervention (n=151)

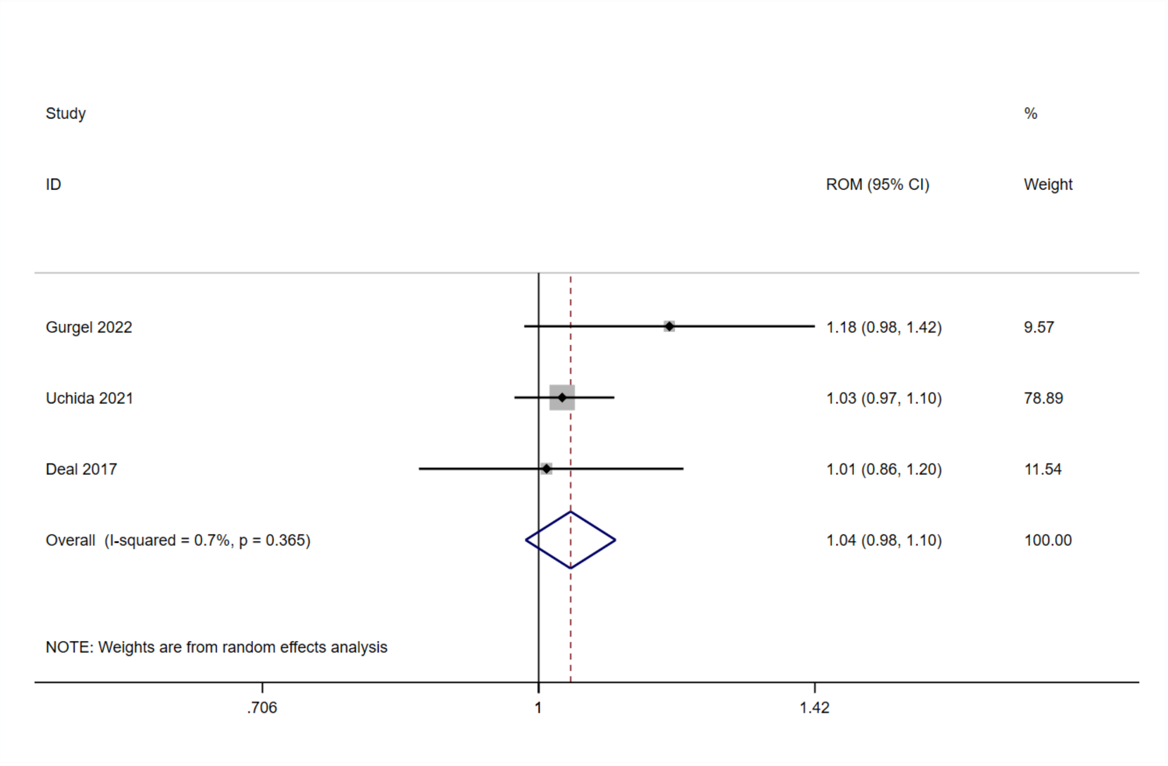

Supplementary Figure S4 Forest plot of changes in memory function test scores before and after hearing intervention (n=1046)

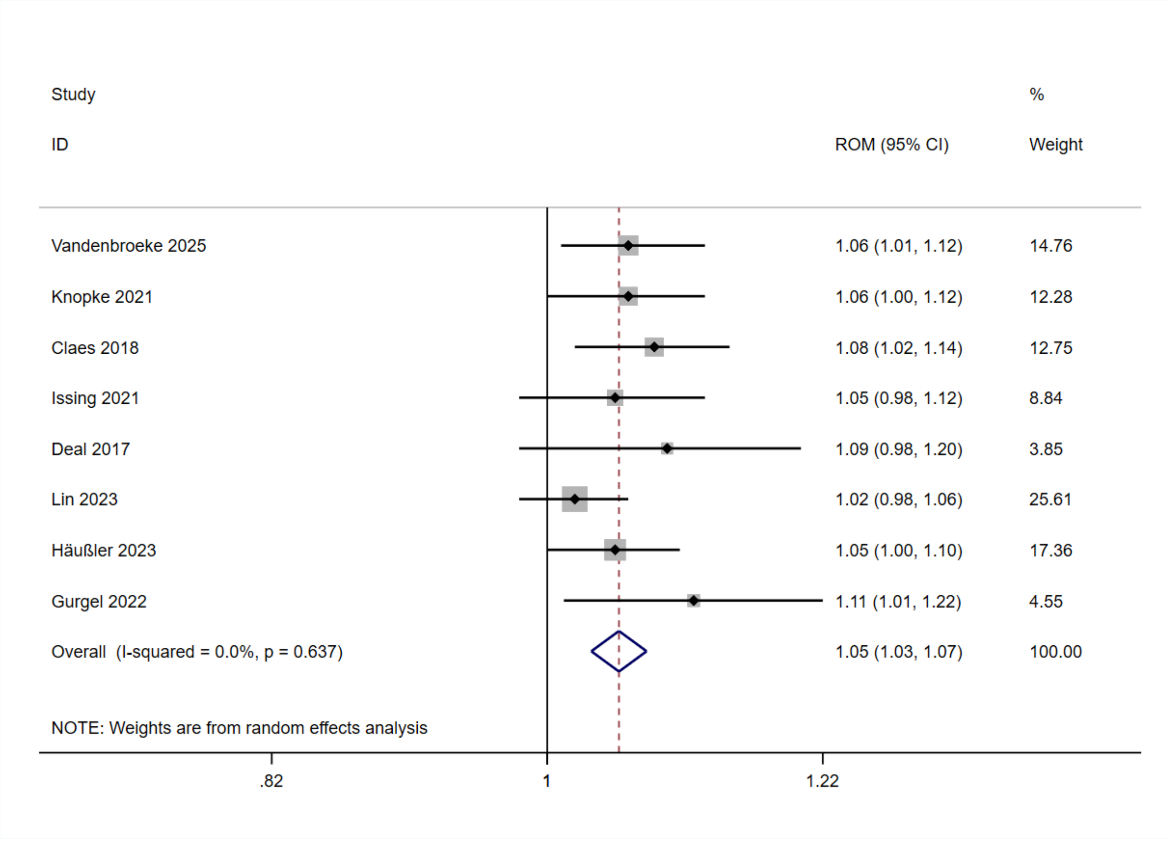

Supplementary Figure S5 Forest plot of changes in attention domain test scores before and after hearing intervention (n=80)

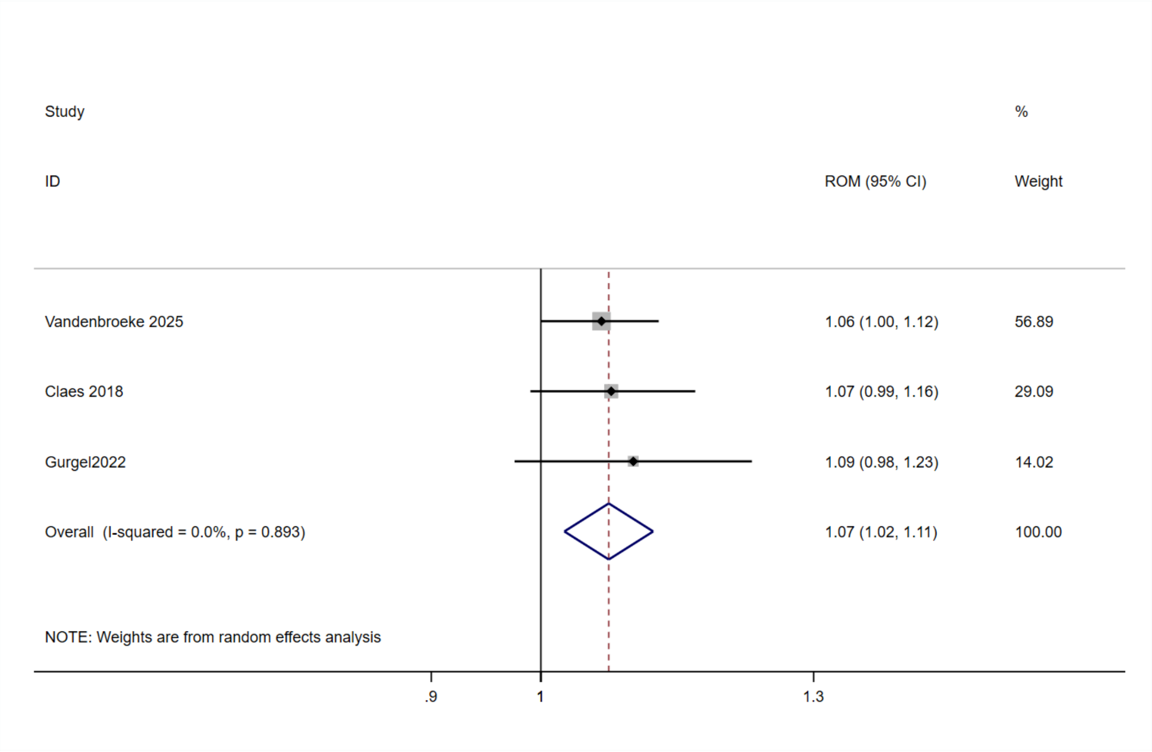

Supplementary Figure S6 Forest plot of changes in information processing domain test scores before and after hearing intervention (n=87)

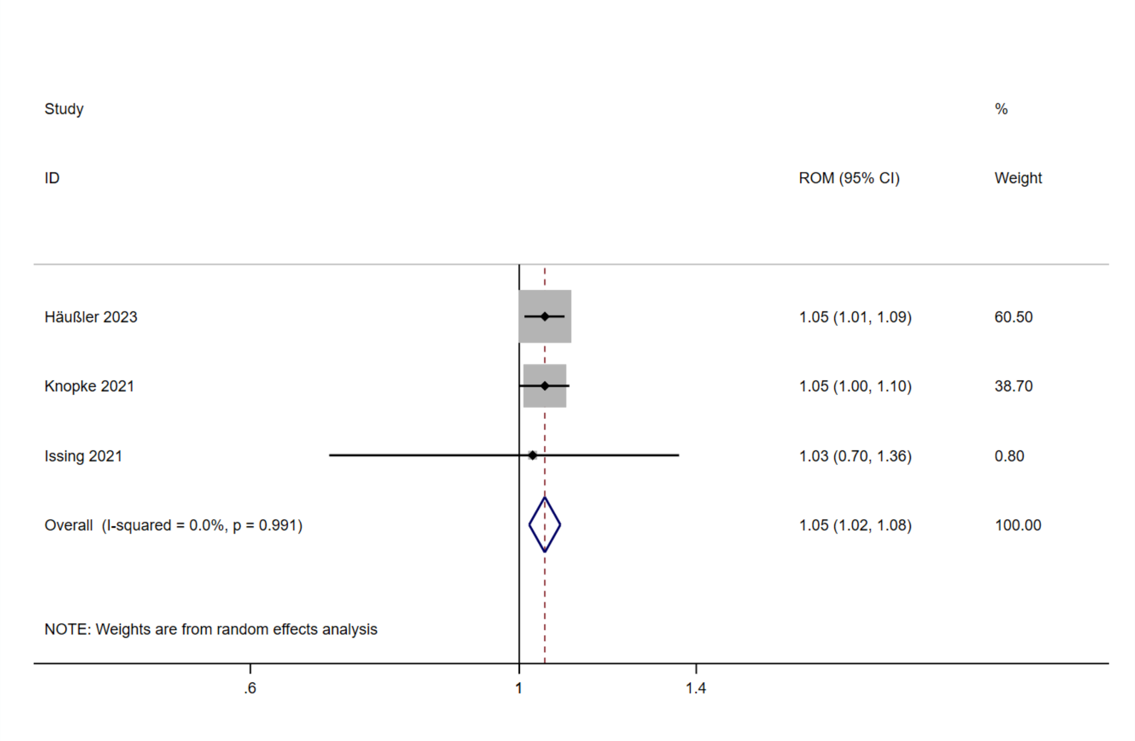

Supplementary Figure S7 Funnel plot of changes in global cognitive test scores before and after hearing intervention

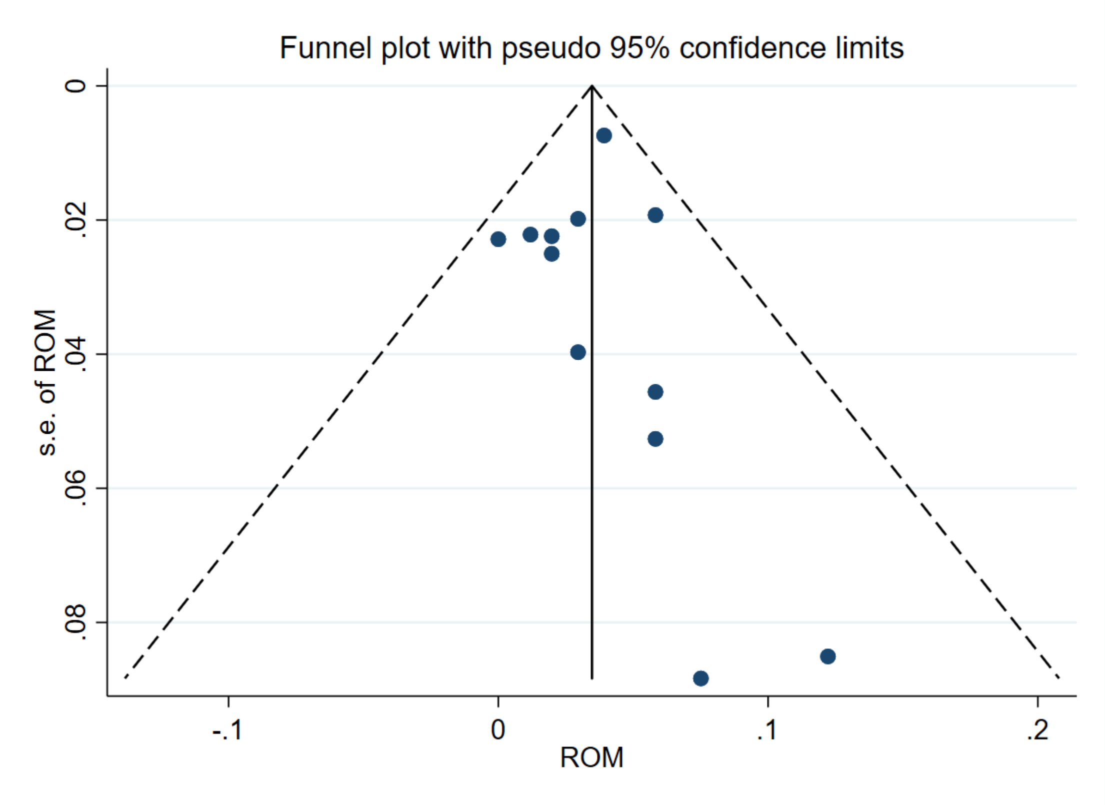

Supplementary Figure S8 Sensitivity analysis of global cognition test score changes before and after hearing intervention

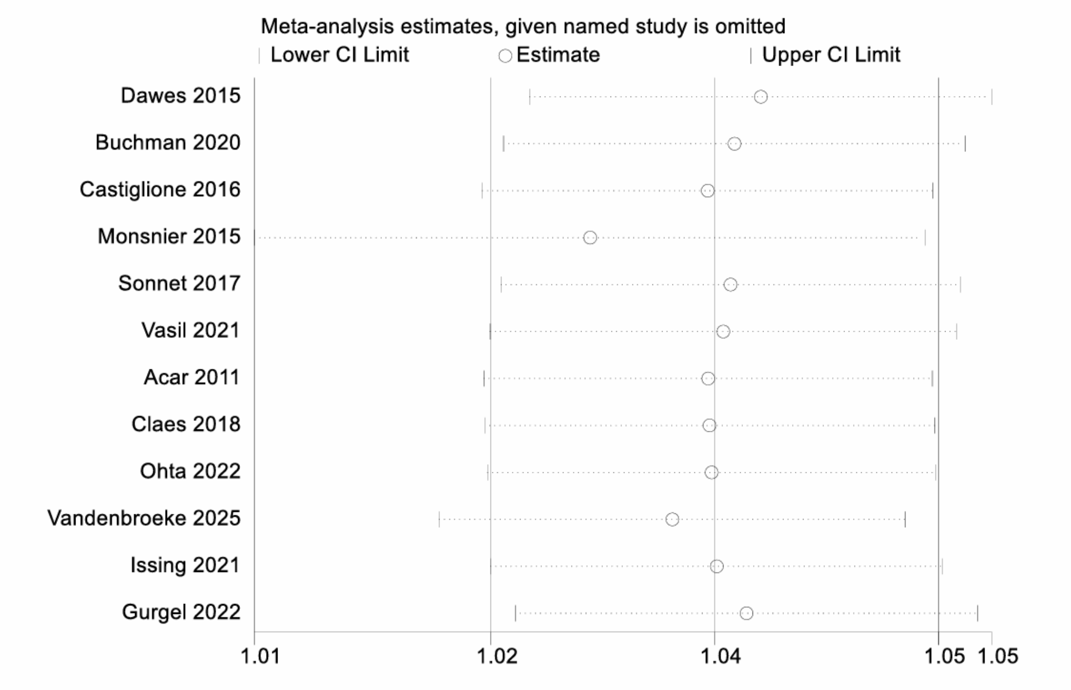

Supplementary Figure S9 Sensitivity analysis of cognition test score changes between intervention group and control group

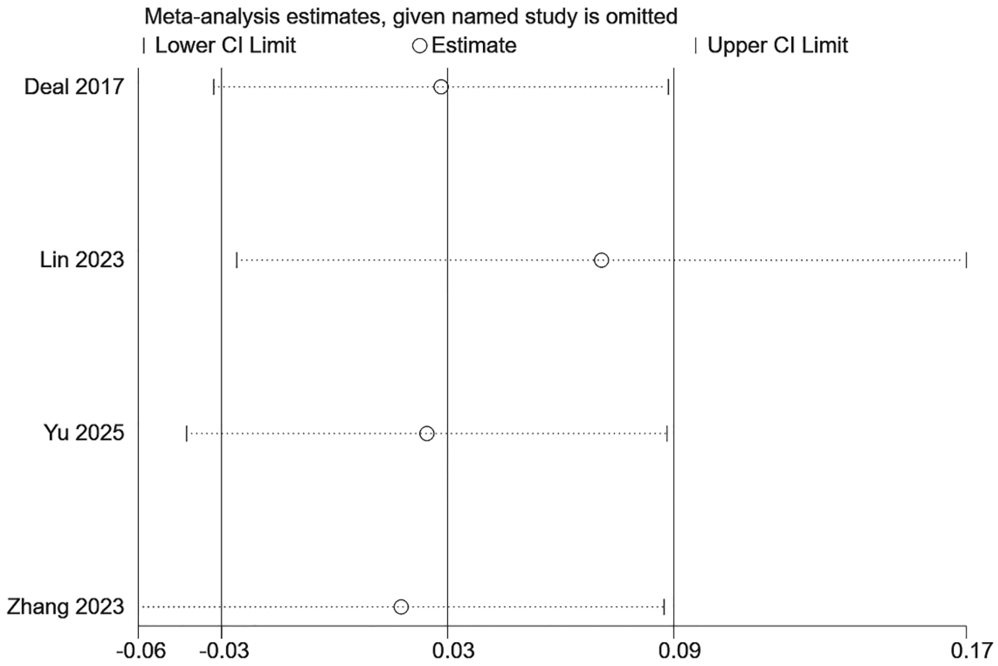

Supplement: Supplementary file 1 [file audiolres-16-00067-s001.zip › audiolres-4143584-supplementary.pdf]
